# High-resolution structure of proIAPP(1–48) fibrils suggests a mechanistic pathway for diabetes-associated IAPP fibril polymorphs

**DOI:** 10.1039/d5cb00228a

**Published:** 2025-10-31

**Authors:** Dylan Valli, Michał Maj

**Affiliations:** a Department of Chemistry—Ångström Laboratory, Uppsala University Uppsala Sweden michal.maj@kemi.uu.se

## Abstract

The human islet amyloid polypeptide (hIAPP) aggregates into amyloid fibrils that contribute to β-cell failure in type 2 diabetes. hIAPP is produced from a 67-residue precursor, proIAPP, but incomplete cleavage by prohormone convertase 2 (PC2) produces the 48-residue intermediate proIAPP(1–48), which accelerates amyloid formation *in vivo*. Here we show that proIAPP(1–48) assembles almost exclusively into a single fibril polymorph. Using cryo-electron microscopy we solved its structure at 3.5 Å resolution and uncovered a P-shaped, C2-symmetric dimer whose backbone and side-chain packing are nearly identical to the disease-associated TW2 polymorph propagated from pancreatic tissue, although with different helical symmetry. All eleven extra N-terminal residues remain disordered but create a weak density around His29. Based on time-averaged density derived from molecular dynamics (MD) simulations, we identified multiple hydrogen(H)-bonding interactions, which may contribute to stabilising the TW2-like fold and explain the peripheral cryo-EM density. These data establish a structural link between defective proIAPP processing and the polymorphic spectrum of islet amyloid and suggest a seeding pathway by which proIAPP(1–48) templates pathogenic architectures that fully processed hIAPP rarely adopts *in vitro*.

## Abstract 2

Structure of misprocessed proIAPP(1–48) shows how processing errors may bias hIAPP toward disease-relevant folds.

## Graphical abstract

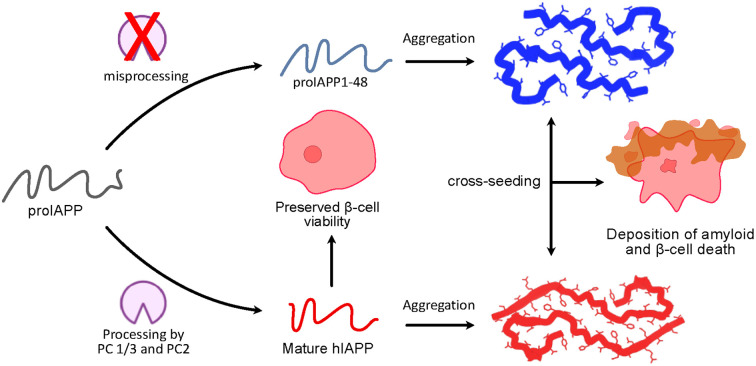

## Introduction

**Fig. 1:**
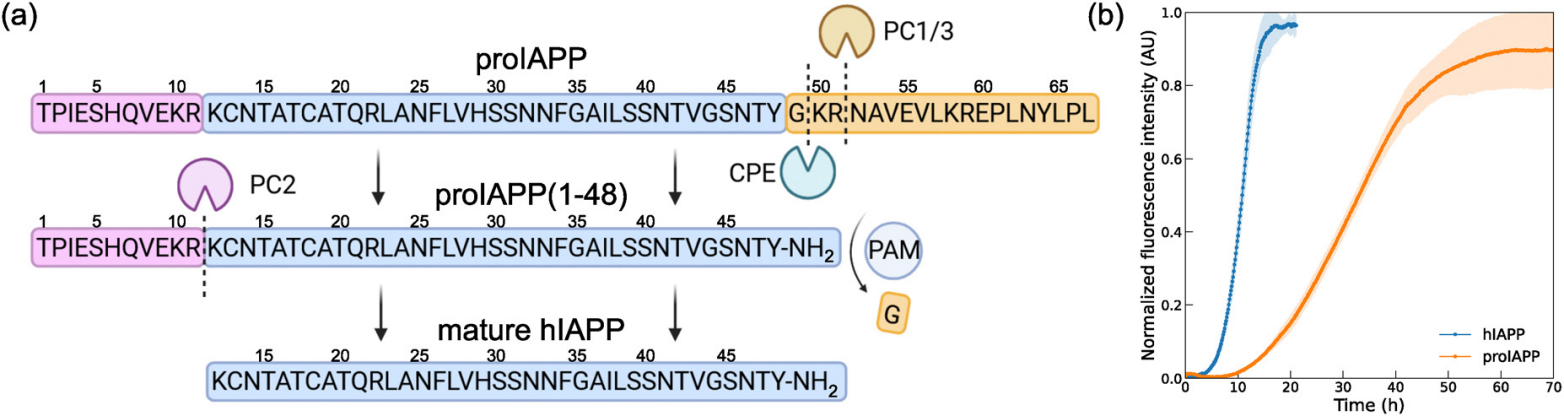
Processing and aggregation kinetics of proIAPP and hIAPP. (a) Schematic of the enzymatic processing of full-length proIAPP, first to the proIAPP(1–48) intermediate and then to mature hIAPP. (b) Aggregation kinetics of proIAPP(1–48) and mature hIAPP monitored by ThT fluorescence. The data reveal a longer lag phase and a slower aggregation rate for proIAPP(1–48) compared to mature hIAPP.

The formation of amyloids in the pancreatic islets is one of the key pathological features of type 2 diabetes.^1,2^ These amyloids are predominantly composed of the human islet amyloid polypeptide (hIAPP), a 37-amino-acid hormone co-secreted with insulin from the pancreatic β-cells.^3–5^ The secretory granules of the β-cells are the primary site for processing both proinsulin and proIAPP.^6,7^ As shown in Fig. 1a, IAPP is synthesized as a 67-residue prohormone with mature hIAPP located in the central segment. The first cleavage event occurs at the C-terminus, mediated by PC1/3, followed by cleavage at the N-terminus by PC2. However, under the metabolic stress associated with diabetes this cleavage process is impaired.^8^ In particular, reduced PC2 function can lead to incomplete removal of the 11-residue N-terminal prosegment, resulting in a truncated intermediate known as proIAPP(1–48). Biochemical studies show that this N-terminally extended intermediate is amyloidogenic, cross-seeds mature hIAPP, and is cytotoxic in β-cell models.^9^

A substantial evidence implicates proIAPP(1–48) as a key initiator of amyloid aggregation. In engineered β-cell lines, for instance, amyloid-like deposits form predominantly when PC2 expression is impaired, and these intracellular deposits show strong immunoreactivity for proIAPP.^10^ Cells with incomplete processing showed reduced viability and signs of toxicity associated with the deposits. Similarly, in transgenic mice overexpressing hIAPP, immuno-electron microscopy has confirmed that the initial intracellular fibrils consist of unprocessed proIAPP.^11^ Furthermore, partial or complete knockout of PC2 causes an increase in amyloid deposition. This outcome correlated with higher levels of proIAPP(1–48) in islets and the circulation. In fact, proIAPP(1–48) has been directly measured in human blood, confirming that the misprocessed peptide is secreted into the circulation. An elevated proIAPP(1–48) to hIAPP ratio was also observed in individuals with type 1 diabetes and recipients of islet transplants.^12^ In human islet grafts transplanted into mice, larger intracellular deposits were seen, which had only patchy proIAPP immunolabeling, suggesting that as amyloid grows, the initial proIAPP core becomes diluted by further addition of mature IAPP. A proposed mechanism for how these seeds might form *in vivo* involves interaction of the N-terminal extension of proIAPP(1–48) with heparan sulfate proteoglycans (HSPGs) in the extracellular matrix.^13^

The important question is how seeding by proIAPP(1–48) influences the final amyloid fibril structure. Recent advances in cryo-electron microscopy (cryo-EM) have revealed that hIAPP fibrils are polymorphic, adopting multiple distinct folds which may differ in stability, seeding propensity, and cytotoxicity.^14^ Most importantly, it has been observed that the amyloid structures seeded by fibrils extracted from the human pancreatic tissue are drastically different from those observed in any studied conditions *in vitro*.^15^ This implies that cellular factors bias assembly toward specific polymorphs.

To understand the role of proIAPP(1–48) and its potential impact on fibril polymorphism it is key to solve the structure of fibrils formed by this intermediate. In this study, we apply cryo-EM and report the first structure of proIAPP(1–48) fibrils. We find that proIAPP(1–48) assembles almost exclusively into a single, structurally homogeneous polymorph. This finding is particularly interesting, since amyloid fibrils grown *in vitro* are typically highly polymorphic. Moreover, its fold closely matches an *ex vivo* hIAPP polymorph obtained by seeding with fibrils from human pancreatic tissue.^15^ The assignment of elusive regions in density maps is supported by extensive molecular dynamics (MD) simulations of the fibril structure, highlighting the role of the N-terminus in stabilizing the assembly. These findings suggest a link between defective proIAPP processing and the polymorphic spectrum of islet amyloid and support a seeding pathway in which misprocessed proIAPP(1–48) templates pathogenic architectures that mature hIAPP rarely adopts *in vitro*.

## Results and discussion

### proIAPP(1–48) exhibits slower aggregation kinetics and assembles into one dominant polymorph

**Fig. 2:**
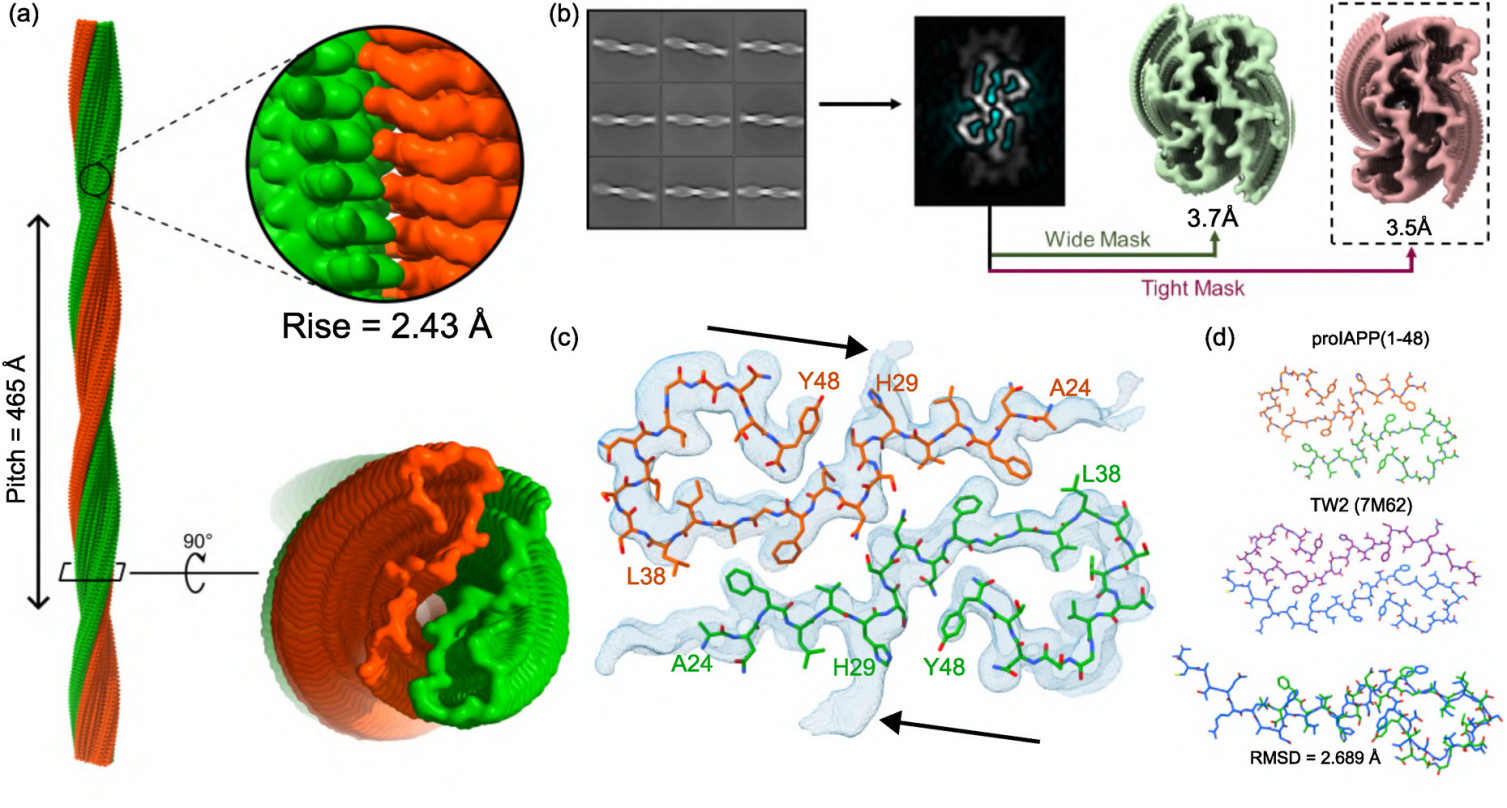
Cryo-EM reconstruction and atomic model of proIAPP(1–48) fibrils. (a) The overall fibril structure consists of two intertwined, P-shaped protofilaments (green and orange) with C2 symmetry. The fibril exhibits a helical pitch of 465 Å and a helical rise of 2.43 Å per subunit. (b) Overview of the cryo-EM helical reconstruction workflow. Representative 2D class averages were used to generate a 3D model. Final refinement using a wide mask (including peripheral density) yielded a 3.7 Å map, while a tight mask focused on the ordered core produced the final 3.5 Å resolution map. (c) A close-up view of the refined atomic model (stick representation) fitted into the cryo-EM density map (blue mesh). The arrows indicate the unresolved extra density. (d) Structural comparison of the proIAPP(1–48) fibril fold (top) with the previously reported *ex vivo* TW2 polymorph of mature hIAPP (middle). A structural alignment (bottom) shows the two folds are nearly identical, with a backbone root-mean-square deviation (RMSD) of 2.689 Å. The solved core of the proIAPP(1–48) structure corresponds to residues 24–48 of the full sequence, resulting in a shorter visible backbone compared to the hIAPP structure.

**Fig. 3:**
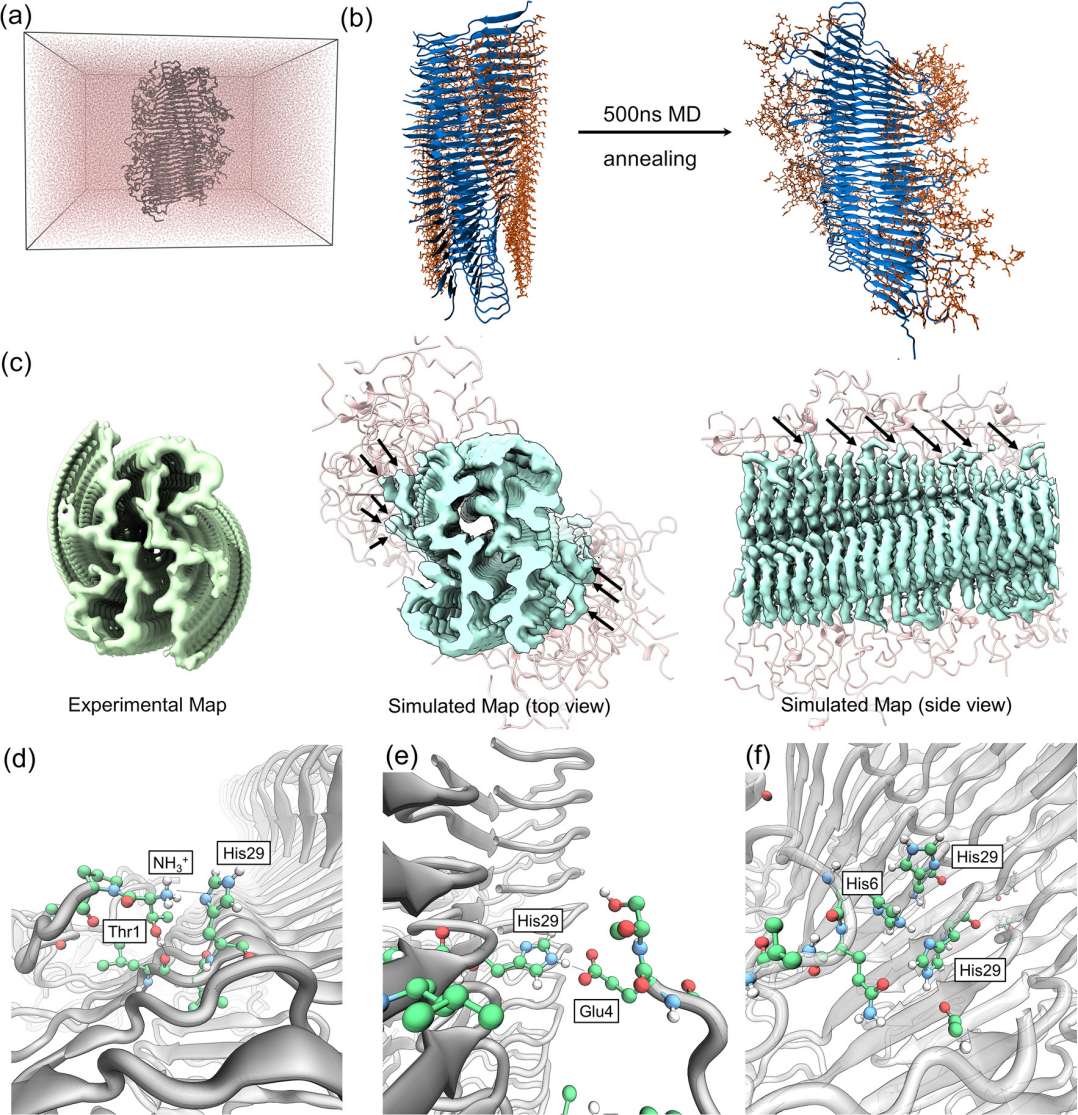
MD simulations reveal N-terminal interactions with the fibril core (a) simulation setup: the full-length proIAPP(1–48) fibril, composed of 25 layers (50 peptide chains), centered within a rectangular periodic boundary box. (b) Conformational sampling of the N-terminus: the initial fibril model with regular N-termini (left, orange) was subjected to 500 ns of MD simulation with cyclic annealing. The final snapshot (right) shows the N-terminal residues exploring the space around the fibril core (blue). (c) Comparison of experimental and simulated cryo-EM densities: Left: experimental cryo-EM density map, showing weak peripheral density surrounding the well-ordered fibril core. Middle (top view) and Right (side view): time-averaged density map derived from MD simulations (cyan) superimposed on the fibril atomic model. The peripheral density lobes (indicated by black arrows) in the simulated map agree well with the experimental density. (d)–(f) Detailed views of key interactions between N-terminal residues and the fibril core identified through MD simulations. The fibril backbone is shown in gray cartoon.

We synthesize proIAPP(1–48) and compare its aggregation profiles to mature hIAPP using a Thioflavin-T (ThT) fluorescence assay, with results presented in Fig. 1b. Both peptides were prepared in triplicates at 100 μM in 10 mM HEPES buffer. The proIAPP(1–48) sample displayed a visibly longer lag phase (10.7 ± 0.2 hours *vs*. 4.3 ± 0.1 hours), and a slower half-time to reach 50% of the final signal intensity (*t*_50_), which was estimated to be 33.0 ± 1.3 hours in proIAPP(1–48) *vs*. 10.8 ± 0.3 hours in WT. The slightly lower end-point intensity for proIAPP(1–48) is attributed to differences in binding affinity of the dye to the fibril products. Both reactions were allowed to proceed for one week, after which the samples were deposited on grids for cryo-EM analysis. We processed cryo-EM data following standard helical reconstruction protocols in RELION5.^16,17^ We acquired 12 061 micrographs, of which approximately one-third were excluded after CTF refinement as they failed to meet the required maximum resolution threshold of 5 Å. Particles were manually selected and subjected to 2D classification (Fig. 3a). All the particle population adopted the same helically twisted fibril polymorph. We excluded the distorted, low quality particles in 2D classification which amounted to about 25% of the total number of particles. The high degree of helical order allowed for straightforward stitching of class averages to span the entire cross-over length. We determined the helical twist of approximately −3.6° if the rise is set at 4.75 Å or −1.8° for the half-rise in pseudo-21 screw symmetry. We built an initial three-dimensional (3D) model from the stitched 2D class average and carried out several rounds of 3D classification. Starting with 23 802 particles, 3D classification yielded 3635 high-quality particles, which were then used for final 3D refinement, Bayesian polishing, and post-processing. The classification and refinement results are shown in Fig. 2b. 3D refinement supported pseudo-21 screw symmetry as the correct helical symmetry, and revealed a compact fibril core flanked by weaker peripheral density, appearing as a closed ring in projection. To refine the map, we applied two masking strategies. A broad mask, including both the core and peripheral regions, produced a reconstruction at 3.7 Å resolution; however, the peripheral density remained featureless, indicating disorder. Using a narrower mask focused exclusively on the continuous core density yielded a 3.5 Å resolution with well-resolved side-chains. The refinement employed pseudo-21 screw symmetry, with a rise of 2.4 Å and a twist of 178.2°. We built the atomic model in Coot,^18^ followed by real-space refinement in PHENIX.^19^ Key data acquisition parameters, helical parameters (rise and twist), and model-building statistics are summarized in Table 1. Local resolution maps and other processing details are provided in the Supplementary Information. The assignment of the masked peripheral density with molecular-dynamics (MD) simulations is examined in a later section. Residues are numbered by the proIAPP(1–48) sequence throughout.

Cryo-EM data-collection, refinement and validation statistics

**Table:** 

Data collection and processing	
Magnification	105 000
Voltage (kV)	300
Electron exposure (e^−^ Å^−2^)	39
Defocus range (μm)	−0.8 to −2.4
Pixel size (Å)	0.825
Symmetry imposed	C2
Twist (°)	178.2
Rise (Å)	2.4
Initial particle images (no.)	23 802
Final particle images (no.)	3 635
Map resolution (Å)	3.5

**Table:** 

Refinement	
Initial model used (PDB)	*de novo*
Model resolution (Å)	3.5
FSC threshold	0.143
Map-sharpening *B* factor (Å^2^)	−117

**Table:** 

Model composition	
Non-hydrogen atoms	1850
Protein residues	250
Ligands	10

**Table:** 

*B* factors (Å^2^)	
Protein	109.26
Ligand	105.10

**Table:** 

R.m.s.d.	
Bond lengths (Å)	0.003
Bond angles (°)	0.618

**Table:** 

Validation	
MolProbity score	1.32
Clashscore	6
Rotamer outliers (%)	0

**Table:** 

Ramachandran plot	
Favoured (%)	100
Allowed (%)	0
Disallowed (%)	0

### Structural model of proIAPP(1–48) shows striking similarity to an *ex vivo* polymorph of hIAPP

The refined cryo-EM map, shown in 2(c), reveals a two P-shaped protofilament fibril structure with C2 rotational symmetry. Our atomic model was built into the well-resolved amyloid core, which comprises residues Ala24 to Tyr48 of each peptide chain. The preceding N-terminal residues (1–23), including the 11-residue pro-segment, are not visible in the map, which indicates that they remain conformationally flexible. The absence of density in the N-terminal region is typical of IAPP fibrils.^14,20–22^ but it is notable here that the extra pro-sequence residues do not integrate into the ordered fibril core.

Each protofilament adopts a cross-β arrangement: one β-sheet resides in the FGAIL region (Phe34–Leu38), and the other spans Ala24–Val28. The interface between the protofilaments is stabilized by hydrophobic interactions involving Phe26, Val28, Phe34, and Leu38. Two asparagine ladders, at positions 25 and 46, provide additional stabilization *via* H-bonds, which is a common motif in amyloid fibrils.^23^ Tyr48 follows a short C-terminal loop that projects outward from the fibril surface.

Remarkably, this proIAPP(1–48) fold is nearly identical to the TW2 polymorph, a structure previously determined for hIAPP fibrils propagated from human pancreatic tissue.^15^ As shown in Fig. 2d, the backbone of our model overlays closely with the TW2 structure, with a root-mean-square deviation (RMSD) of only 2.689 Å. However, despite the shared fold, the two fibrils display different helical parameters. The proIAPP(1–48) fibril is more tightly twisted, with a twist of 178.2° and a rise of 2.4 Å (assuming pseudo-21 symmetry), compared to the TW2 polymorph (178.42° twist, 4.80 Å rise). Despite the different helical parameters, templating is expected because nucleation proceeds *via* small assemblies in which twist is not yet fixed; as shown below, transient contacts from the 11-residue N-terminal prosegment can bias inter-protofilament geometry and thus the final twist.

The preference for this P-shaped, TW2-like fold is significant because mature hIAPP typically forms the so-called double-S polymorph *in vitro* (Fig. S7). A key difference is that the double-S structure features a bend after Phe34 that orients His29 far from the C-terminal Tyr48. In our proIAPP(1–48) model, these two residues are positioned in close proximity. Considering the weak peripheral densities we observe near His29, we propose that interactions between the flexible N-terminal extension and this region of the fibril core help stabilize the P-shaped conformation, thus favoring this rare architecture. To test this hypothesis and identify the specific atomic contacts responsible for this effect, we performed all-atom MD simulations of the fibril structure.

### Molecular dynamics of proIAPP(1–48) fibrils uncover unique interactions between the N-terminus and its fibril core

To investigate the origin of the weak peripheral densities seen in our cryo-EM map, we performed MD simulations of the full-length proIAPP(1–48) fibril. Simulations were carried out in GROMACS 2025.1^24,25^ using GROMOS54a7 force field^26^ and SPC^27^ water, a combination widely employed for amyloid peptides and explicitly benchmarked for Aβ/IAPP systems.^28–30^ More detailed description of the simulations is given in the SI. We used the refined cryo-EM structure (residues 24–48) as a rigid core and appended the missing N-terminal residues (1–23) in Coot.^18^ We then constructed a 25-layer model (50 peptide chains), aligning the fibril along the short dimension of a rectangular periodic box. We select such irregular box to model an infinitely long fibril, while leaving space for the flanking side chains to fully unfold away from its periodic image. The final system comprised 23 100 atoms (462 atoms per peptide). After heating to 500 K to randomize the flexible N-terminal region, we cooled the system over 100 ns and performed cyclic annealing between 300 K and 350 K for 500 ns.

From the resulting trajectory, we generated time-averaged simulated density maps using the MDFF sim module. These maps represent the most frequently occupied positions of the N-terminal residues, while the highly flexible structures average out to zero. The comparison with the experimental density (Fig. 3c) reveals peripheral density lobes consistent with recurrent contacts between N-terminal residues and His29. To probe these interactions in detail, we extracted four-peptide, double-layer fragments from the central region of the fibril, excluding four layers from each end to minimize edge effects. We evaluated conformational similarity by pairwise RMSD calculations on heavy side-chain atoms and clustered the structures with GROMOS using a 1.0 nm cut-off.^31^ The six most populated clusters are shown in Fig. S7 in the SI.

The most dominant and stable interaction observed involved the terminal amine forming a H-bond with His29 (Fig. 3d). This interaction was frequently accompanied by a secondary H-bond between the hydroxyl group of Thr1 and the backbone carbonyl of Leu27. Although this primary contact was transient, it consistently reformed with different His29 residues on adjacent layers on sub-ns timescales. Similar interactions between protonated amines and stacked histidine residues have been reported in Rh family transporters.^32,33^

We also observed intermittent hydrogen bonds between His29 and the side chain of Glu4 (Fig. 3e), an interaction unique to proIAPP(1–48) as mature hIAPP lacks glutamate residues. Finally, a less frequent but stable three-histidine cluster was seen, where His6 would coordinate with two His29 residues from neighboring layers (Fig. 3f). These triads persisted for extended timescales, although the majority of transient conformations eventually reverted to the dominant motif shown in Fig. 3d. Longer simulations or enhanced sampling methods may clarify whether the His6-based triads converge to other, more prevalent states. Together, these transient interactions explain the weak, averaged-out density features observed experimentally and confirm that the N-terminal extension exhibits strong attractive forces around its His29 residue. Together, these recurrent yet short-lived contacts rationalize the weak peripheral density around His29 in the cryo-EM map (Fig. 3c) and provide a mechanism by which the flexible N-terminus can modulate inter-protofilament geometry, and hence helical twist, without altering the core β-sheet registry; because nucleation begins from small assemblies in which twist is not yet rigidly fixed, templating of the core fold by proIAPP(1–48) remains compatible with the modest twist differences observed at bulk scale.

## Conclusions

In this study, we determined the high-resolution cryo-EM structure of fibrils formed by proIAPP(1–48), an intermediate product of impaired prohormone processing linked to type 2 diabetes. We found that proIAPP(1–48) assembles into a single, structurally homogeneous polymorph whose fold is nearly identical to the disease-associated TW2 polymorph propagated from human pancreatic tissue. Our MD simulations provide a mechanistic explanation for this finding, revealing that the flexible N-terminal extension stabilizes this specific pathogenic architecture through transient interactions with the fibril core, particularly around the His29 residue. Future work should focus on experimentally validating the role of N-terminus in polymorph selection and stabilization of disease-relevant structures, which may guide the development of new strategies to prevent the formation of toxic amyloid species in type 2 diabetes.

## Author contributions

Dylan Valli: data curation, investigation, validation, visualization, writing – review & editing. Michał‚ Maj: conceptualization, data curation, formal analysis, funding acquisition, project administration, supervision, validation, writing – original draft.

## Conflicts of interest

There are no conflicts to declare.

## Supplementary Material

CB-007-D5CB00228A-s001

## Data Availability

The data supporting this article have been included as part of the supplementary information (SI). Supplementary information:  (i) peptide synthesis and purification and characterization procedures; (ii) full molecular-dynamics setup and parameters; and (iii) Supplementary Fig. S1–S7 present cryo-EM map orientations, local-resolution map, FSC curves, residue-type schematic, EMReady pre/post comparison of the map, comparison with the TW3 fold, and GROMOS clustering of MD fragments. Structural data have been deposited into the Worldwide Protein Data Bank (wwPDB) and the Electron Microscopy Data Bank (EMDB) with the following accession codes: PDB 9SL2; and EMD-54982. See DOI: https://doi.org/10.1039/d5cb00228a.
