# Supplementary material for: High-resolution structure of proIAPP(1–48) fibrils suggests a mechanistic pathway for diabetes-associated IAPP fibril polymorphs
Source: RSC Chem Biol. 2025 Oct 31;7(1):38–43. doi: 10.1039/d5cb00228a (PMC12590159; doi:10.1039/d5cb00228a)
Supplement: CB-007-D5CB00228A-s001 [file CB-007-D5CB00228A-s001.pdf]

This journal is © The Royal Society of Chemistry 2025

Electronic Supplementary Information for: High-Resolution Structure of proIAPP(1–48) Fibrils Suggests a Mechanistic Pathway for Diabetes-Associated IAPP Fibril Polymorphs

Dylan Valli[a] and Michał Maj[a,*]

[a]Department of Chemistry, Uppsala University, Uppsala, Sweden
*E-mail: michal.maj@kemi.uu.se

Peptide synthesis and purification

proIAPP(1-48) was synthesized on a 0.1 mmol scale using standard 9-fluorenylmethyloxy-carbonyl (Fmoc) chemistry on a Biotage Initiator+ Alstra peptide synthesizer. Tentagel R RAM resin was used to obtain the C-terminal amide. Pseudoproline dipeptide derivates were incorporated to facilitate the coupling of difficult regions according to previous IAPP synthesis protocols.[1] The final product was cleaved from the resin using a cocktail of 95% TFA, 2.5% water, and 2.5% triisopropylsilane for 3 hours. The crude peptide was precipitated with cold diethyl ether, redissolved in H2O, and lyophilized. The peptide was oxidized for 24 hours to form disulfide bridge between Cys2 and Cys7 by dissolving in 60% DMSO. Purification of the peptide was performed using reverse-phase HPLC (Isera C18 preparative column, 250 x 20 mm), with a gradient elution composed of buffer A (100% H2O with 0.045% HCl) and buffer B (80% acetonitrile with 0.045% HCl. After lyophilization the peptide was resuspended in 50 % hexafluoroisopropanol and 20 % acetic acid and purified a second time. The mass of the peptide was confirmed using MALDI-FTICR-MS. The pure peptide was monomerized by resuspending in 100 % HFIP and filtering through a 0.22 μ m filter and the concentration was determined using absorbance at 280 nm using the extinction coefficient of tyrosine (1490 $M^{-1}cm^{-1}$). The peptide was aliquoted and lyophilized to remove traces of HFIP and stored dry at -20 °C.

Molecular dynamics simulations

The starting fibril model was built from the refined cryo-EM structure of the ordered core (Ala24–Tyr48) and extended to full-length proIAPP(1–48) by adding the missing N-terminal residues 1–23 in Coot. A two-protofilament assembly was generated and stacked to 25 layers (50 peptide chains total) to create a periodic segment. The fibril axis was aligned with a box vector, and a rectangular periodic box was chosen such that the box dimension parallel to the fibril axis matched the helical rise per periodic repeat to ensure seamless propagation under PBC, while the two orthogonal dimensions provided at least 6.0 nm solvent padding to minimize self-interaction of the flanking chains.

Standard protonation states at pH 7.4 were used (N-terminus $NH3^{+}$), amidated C-terminus. The protein was described with the GROMOS54a7 force field[2] with SPC water model[3]. The system charge was neutralized with Cl^{-}. To preserve the cryo-EM-derived core while allowing the N-terminal extension to explore conformational space, we applied harmonic position restraints (1000 kJ mol^{-1} nm^{-2}) to the backbone heavy atoms of residues Ala24–Tyr48 in all chains. Residues 1–23 were unrestrained. Simulations were performed with GROMACS 2025.1[4,5] using the Verlet cut-off scheme, real-space cut-offs of 1.0 nm for both Coulomb and Lennard-Jones interactions (LJ truncated at 1.0 nm), particle-mesh Ewald electrostatics (fourth order; Fourier grid spacing 0.12 nm), and a neighbor list updated every 10 steps. All bonds to hydrogens were constrained with LINCS (order 4), allowing a 2 fs time step. The simulations were conducted in the NVT ensemble with a stochastic velocity-rescale thermostat.[6] The protocol comprised steepest-descents energy minimization of the restrained system, a brief 300 K NVT pre-equilibration (2–5 ns), high-temperature randomization at 500 K for 100 ns to decorrelate the flexible N-termini, controlled cooling from 500 to 300 K over 100 ns, and a 500 ns production run employing periodic simulated-annealing cycles of 300 $\rightarrow$ 350 $\rightarrow$ 300 K with an 8 ns period (4 ns heating, 4 ns cooling).

Supplementary Figures

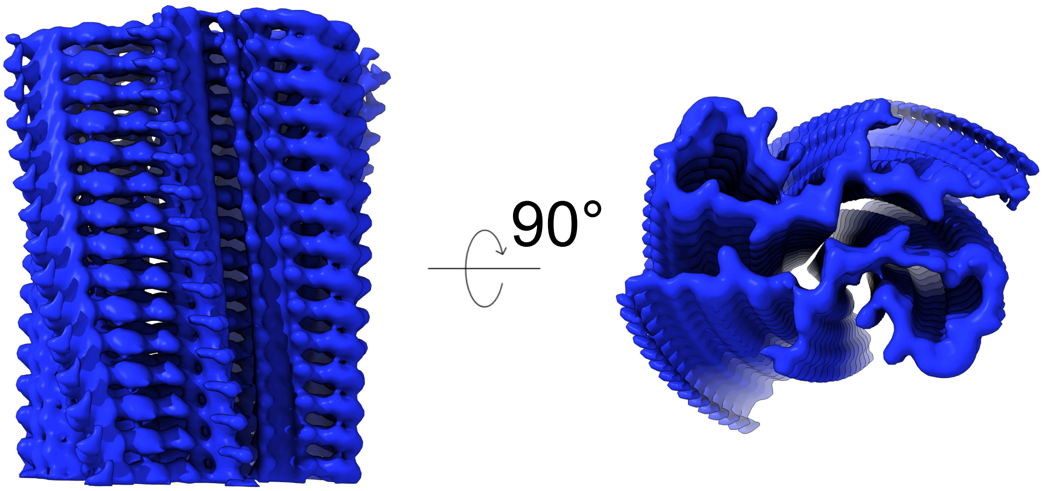

Figure 1 Different orientations of the cryo-EM map.

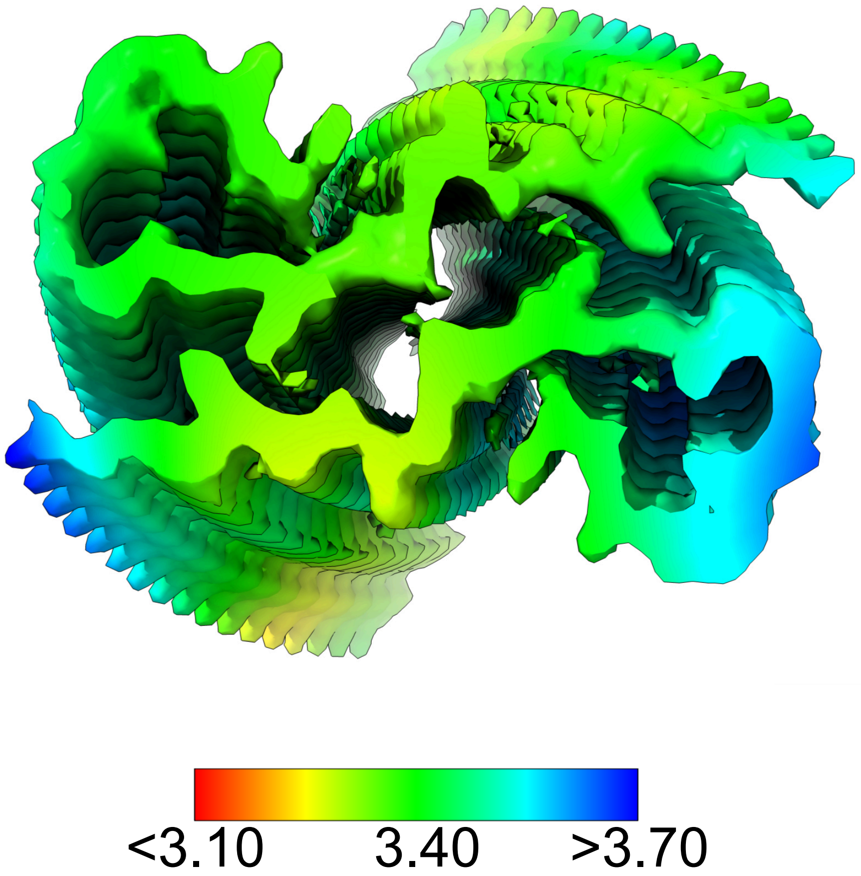

Figure 2 Local resolution estimate computed in RELION.

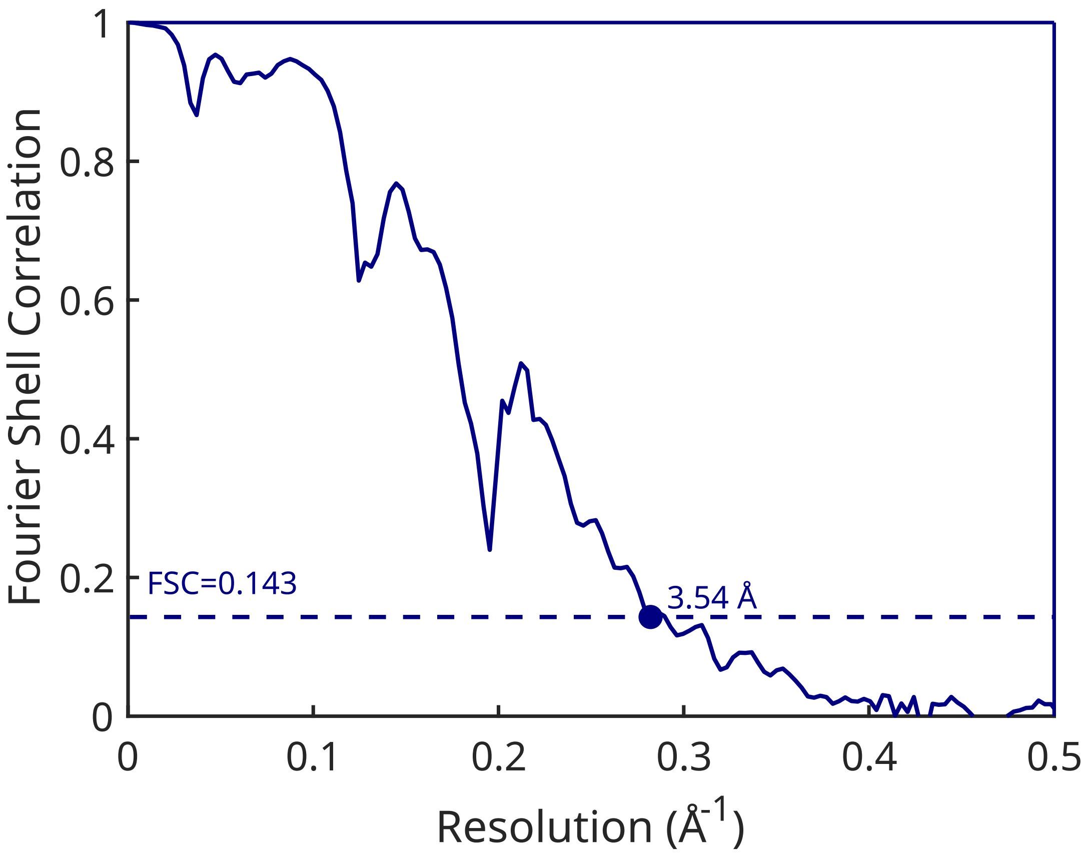

Figure 3 Fourier Shell Correlation curves of the half maps. The resolution at the 0.143 threshold is shown.

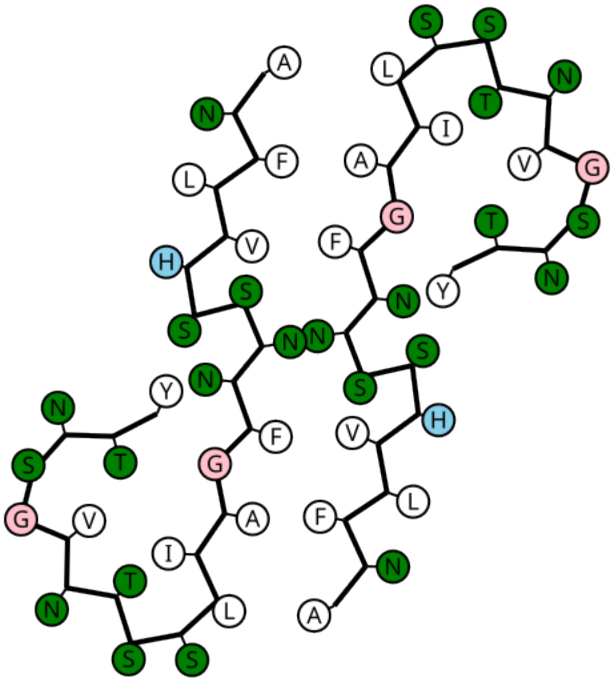

Figure 4 Schematic representation of the proIAPP(1-48) structure using atom2svg[7]. Positively charged residues are colored blue, polar residues are colored green, non-polar residues are colored white, and glycines are colored pink.

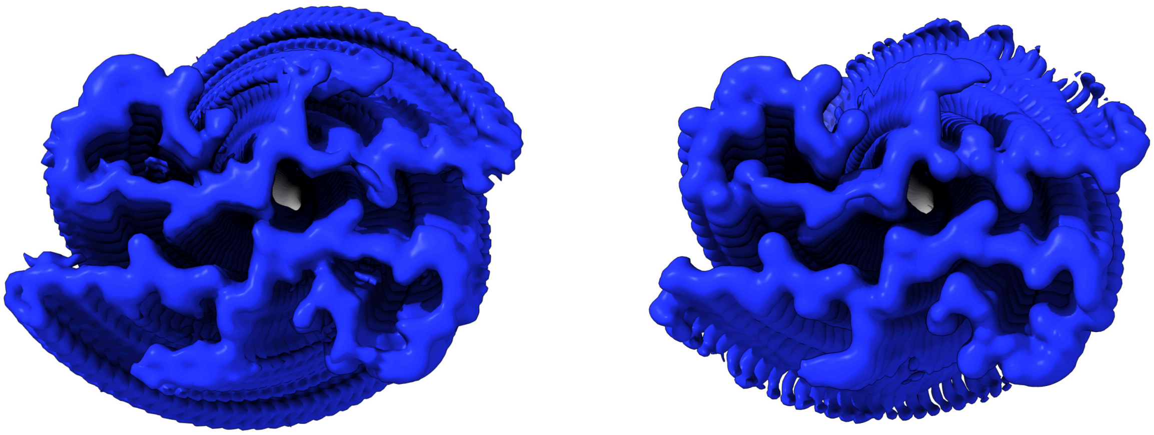

Figure 5 Comparison of the density map before (left) and after (right) processing with EMReady [8]

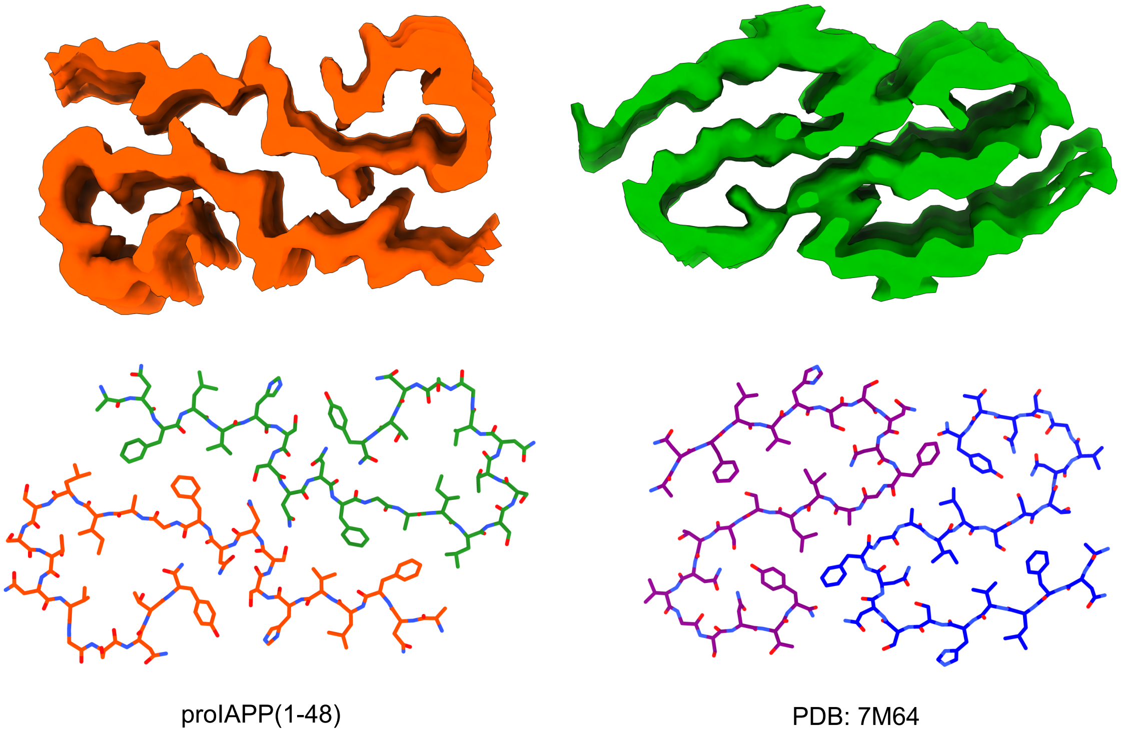

Figure 6 Cryo-EM density maps and atomic models of proIAPP(1-48) (left) and TW3 from Cao et al.[9] (right).

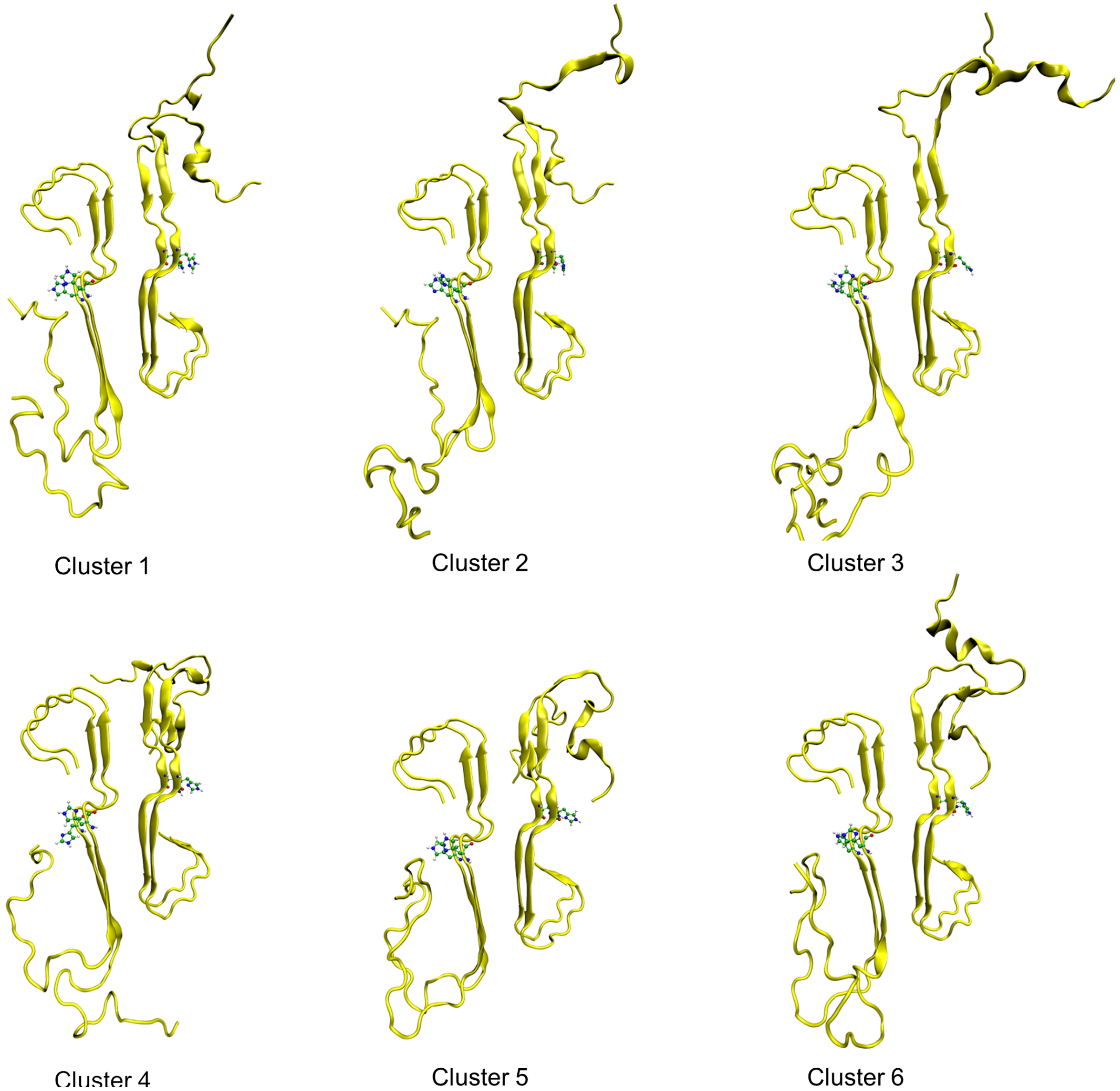

Figure 7 The results of the GROMOS clustering algorithm results showing six most representative double layer segments from the MD simulations.

References

Notes and references

[1] A. Abedini and D. P. Raleigh, *Organic letters*, 2005, **7**, 693–696.

[2] A.-P. E. Kunz, J. R. Allison, D. P. Geerke, B. A. C. Horta, P. H. Hünenberger, S. Riniker, N. Schmid and W. F. van Gunsteren, *J. Comput. Chem.*, 2012, **33**, 340–353.

[3] H. J. C. Berendsen, J. R. Grigera and T. P. Straatsma, *J. Phys. Chem.*, 1987, **91**, 6269–6271.

[4] H. Bekker, H. J. C. Berendsen, E. J. Dijkstra, S. Achterop, R. van Drunen, D. van der Spoel, A. Sijbers, H. Keegstra *et al.*, Physics Computing 92, Singapore, 1993, pp. 252–256.

[5] H. J. C. Berendsen, D. van der Spoel and R. van Drunen, *Computer Physics Communications*, 1995, **91**, 43–56.

[6] G. Bussi, D. Donadio and M. Parrinello, *J. Chem. Phys.*, 2007, **126**, 014101.

[7] T. Nakane, *atom2svg*, `https://gist.github.com/biochem-fan/026ec2f191fee9285424d12fc2b84ce7`, version 210224.

[8] J. He, T. Li and S. Y. Huang, *Improvement of cryo-EM maps by simultaneous local and non-local deep learning*, `https://doi.org/10.1038/s41467-023-39031-1`, 2023.

[9] Q. Cao, D. R. Boyer, M. R. Sawaya, R. Abskharon, L. Saelices, B. A. Nguyen, J. Lu, K. A. Murray, F. Kandeel and D. S. Eisenberg, *Nature structural & molecular biology*, 2021, **28**, 724–730.
